# Efficacy of Two Stabilizers in Nanoemulsions with Whey Proteins and Thyme Essential Oil as Edible Coatings for Zucchini

**DOI:** 10.3390/membranes12030326

**Published:** 2022-03-15

**Authors:** Iulia Bleoanca, Andreea Lanciu, Livia Patrașcu, Alina Ceoromila, Daniela Borda

**Affiliations:** 1 Faculty of Food Science and Engineering, Dunarea de Jos University of Galati, 800201 Galati, Romania; iulia.bleoanca@ugal.ro (I.B.); andreealanciu@yahoo.com (A.L.); 2 Cross-Border Faculty, Dunarea de Jos University of Galati, 111 Domneasca Str., 800201 Galati, Romania; livia.mantoc@ugal.ro (L.P.); alina.cantaragiu@ugal.ro (A.C.)

**Keywords:** *Cucurbita pepo*, fresh perishable vegetables, nanocoatings, active food packaging, shelf-life prolongation, thyme essential oil, guar and arabic gum, polysorbate

## Abstract

Edible coatings are important for horticulture crops preservation and reducing food waste. Application of edible coatings followed by low-temperature storage prolongs the storability, preserves quality, and decreases the overall postharvest losses. This study evaluated the efficacy of two nanoemulsions formulae containing thyme essential oil and whey proteins as coatings for zucchini, with the purpose of extending their shelf-life. The nanoemulsions were rheologically evaluated and the formula with guar and arabic gum mix stabilizer (S) showed a better capacity to restructure after strain compared to the formulae with Tween 20 (T). The S coating material had a better capacity to integrate nanoparticles compared to T. However, when applied on zucchini, T coating was more effective in reducing weight loss showing 16% weight loss compared to 21% in S, after 42 days. At the end of storage at 10 °C, the T-coated zucchini had better firmness (*p* < 0.05) compared with S and both coatings were superior to control (*p* < 0.05). POD (peroxidase) activity was high in peel at the end of storage when also CAT (catalase) showed a sudden increase. On the 42nd day of storage, the highest enzymes activity (CAT, POD, and APX (ascorbate peroxidase)) was present in the S-coated zucchini peel. The most abundant volatile in T coating was α-pinene and 4-carene in S. Sensory analysis showed that T coating delayed the appearance of senescence while S exhibited surface cracks.

## 1. Introduction

Zucchini (*Cucurbita pepo* L.) or summer squash is a very popular soft skin vegetable, rich in antioxidants such as betacarotene, folic acid, vitamins C and E [1]. Ripened zucchini has a firm texture, which softens during storage. Due to its over 90% moisture content, zucchinis are highly perishable vegetables, with an average storage life of 1–2 weeks at 7–10 °C, they are cold-sensitive and susceptible to chilling injury when stored at temperatures below 7 °C [2,3,4]. Simultaneous depletion of antioxidant defense and reactive oxygen species occurrence during zucchini postharvest cold storage [5] leads to their rapid deterioration, visible by browning, firmness loss, and decay [1].

To strengthen the quality during storage, extend shelf-life, and increase market acceptability of raw zucchini, several technologies are available: e.g., vacuum impregnation [6], γ-aminobutyric acid treatment [7], hot water forced convection treatment [8], nitric oxide treatment [5].

An environmentally sustainable solution for shelf-life extension is the application of edible coating (EC) to fresh agricultural products. EC is a thin layer of edible material directly formed on food surface as a coating [9,10] from either polysaccharides, proteins, lipids, or a mix of the above, that can provide a selective barrier to water vapors, oxygen, carbon dioxide, and volatiles, as well as a defense against mechanical disruption of the outer layer of the coated food product without altering the taste and texture of the food. For example, a group of researchers [11] formulated an EC made of casein, chitosan, and carboxymethyl cellulose applied directly on squash slices which produced a 1-log cycle reduction in the counts of aerobic mesophilic bacteria.

Moreover, EC can be functionalized with antimicrobials, antioxidants, or nutraceuticals. Plant essential oils (EO) are widely used for their antioxidant effect and/or large spectrum of action against microorganisms, for example, oregano or clove EOs incorporated into whey protein isolate edible films as natural antimicrobials aimed to enhance the microbial quality of poultry [12]; quince seed mucilage film with oregano or thyme EO extended the shelf life of rainbow trout fillets by 3–11 days during refrigerated storage [13]; carrageenan/chitosan edible films functionalized with natural extracts of Clitoria ternatea, Brassica oleracea, and Ipomea batatas applied to fresh-cut apple pieces [14]; bi-layer of whey protein and xanthan gum with clove applied on tomatoes [15]. Coatings applied on fresh-cut vegetables and fruits, for example, whey proteins and zein thin layer applied on peeled garlic clove [16]; whey protein/pectin edible coating applied on fresh fruit and vegetable [17] were also effective in prolonging shelf-life. Recent reviews have summarized the most important findings on edible coatings with antimicrobials [18] or other active components [19] and underlined the prospects for developing new food applications.

An emerging tool for optimizing the dispersion of EOs’ lipophilic active ingredients in an aqueous media is the use of oil-in-water edible nanoemulsion, where the lipid nanodroplets are stabilized by surfactant molecules dispersed in an aqueous continuous phase [20] to be applied as a coating. Nanoemulsions can be obtained following top-down approaches, through mechanical size reduction techniques such as colloid milling, ultrasonication, and high-pressure homogenization or bottom-up approaches, based on spontaneous self-assembly of surfactants around EOs molecules, like self-emulsification, phase inversion, or solvent demixing [21]. The use of EOs as preservatives in the food industry is limited by their strong flavor, but incorporation in coatings as nanoparticles has the advantage of greater bioactivity, increased diffusion while the impact on sensory properties is diminished in comparison with the non-encapsulated EOs [18].

Nanoemulsions have advantages over solid nanoparticles and liposomes encapsulation methods because they ensure an equal distribution of the hydrophobic components in the hydrophilic matrix and higher stability [18].

The use of nanoemulsions for EC brings advantages in terms of improved homogeneity, increased transparency due to the weak scattering of light by the nanodroplets, improved bioavailability, and stability of EOs’ bioactive compounds, as well as the enhanced released rate from EC matrix [20,22,23]. For example, the safety and quality parameters of fresh-cut Fuji apples along storage time were improved by their coating with nanoemulsion with lemongrass EO [22]. Moreover, in EC obtained from nanoemulsions with active nanoparticles, high specific surface area and improved barrier and scavenging activity [19] was noticed. However, oiling in EC with EOs and sometimes impaired mechanical characteristics was observed [18]. This study aimed to assess the capacity of protein-thyme EO nanoemulsion applied as a coating to extend zucchini’s shelf-life and mutually compare the coatings obtained with two stabilizers to retain the active compounds and thus exert a protective effect on zucchini. Moreover, the coatings’ composition impact on zucchinis’ sensorial and physicochemical properties during storage was evaluated.

## 2. Materials and Methods

### 2.1. Materials

#### 2.1.1. Zucchini

A batch of 36 freshly harvested gray, mature zucchinis (*Cucurbita pepo* L.), *Cuarzo* variety, were bought from a local market (Galati, Romania). They were selected based on their similar size (255.37 ± 37.28 g), color, and shape, without any bruises or other visible injuries. The zucchinis were thoroughly washed with tap water, rinsed with Chicco disinfectant for general use, recommended for fruit and vegetables (Artsana SpA, Busnago, Italy), followed by tap water rinsing. The zucchinis were dried at room temperature, 22 ± 1 °C for 30 min. For the experiments, zucchinis were randomly selected for replicates and treated independently.

#### 2.1.2. Coating Materials

Whey protein concentrate ProMilk 802FB was kindly offered by KUK-Romania (composition on the dry-weight basis: protein content 77%, 1% total fat, 11% lactose, 2.9% total ash, 5% moisture). Anhydrous glycerol (98% purity) was purchased from Redox SRL (Bucharest, Romania). Tween 20 was purchased from Sigma-Aldrich (Bucharest, Romania). Guar and arabic gum mix Satiagel RP1730 was also kindly offered by KUK-Romania. Thyme (*Thymus vulgaris*) EO, kindly provided by SC Hofigal SRL (Bucharest, Romania).

### 2.2. Methods

#### 2.2.1. Preparation of the Coating Nanoemulsion

WPC powder 8.0% (*w*/*w*) was dispersed into ultrapure water under continuous magnetic stirring (180 rpm, 15 min). Thermal treatment was applied to unfold the whey proteins using a thermostatic water bath at 80 ± 0.5 °C for 35 min once the temperature inside the sample has reached the indicated temperature. Immediately after finishing the thermal treatment, the samples were cooled in iced-water to stop the thermal effect. Anhydrous glycerol 8.0% (*w*/*w*) was added as a plasticizer to the resulting coating mixture. This formula was previously optimized by Bleoanca et al. [24].

The samples were divided in half and two stabilizers were tested, one using Tween 20 (T) in a concentration of 0.9% (*w*/*w*) [24] and the other half using 0.18% (*w*/*w*) mix of gums (S), concentration previously optimized by our group (data not shown). Thyme (*Thymus vulgaris*) EO was added in a concentration of 2.5% (*w*/*w*) in all samples, considering the results of previous tests performed by our research group. This particular EO was chosen due to its high content of efficient antimicrobial terpenes [24].

For obtaining a stable and homogenous nanoemulsion the mix was further sonicated (35% amplitude, 3 min) with Sonopuls HD3100 (Bandelin, Germany) equipment, using an 8 mm diameter sonication probe. The sonication tip was maintained immersed 1 cm below the suspension surface and the temperature of the coating nanoemulsion was maintained constant at 23 ± 1 °C by placing the falcons in an iced water bath. The pH was adjusted to 7.0 using NaOH 0.5 N [24].

#### 2.2.2. Characterization of Coating Nanoemulsion

##### Rheological Measurements

Rheological properties were measured with a controlled stress rheometer AR2000ex (TA Instruments LTD, New Castle, DE, USA), using a double-gap concentric cylinder geometry (inner radius of 32 mm, the outer radius of 35.03 mm, cylinder length of 54.95 mm, and gap of 0.5 mm). The temperature was maintained at 30 °C by the Peltier Jacket system. During experiments, the measurement system was covered with a special cover plate device in order to avoid evaporation. Rheological behavior of samples was observed during small amplitude oscillatory strain sweep (% strain = 0.1–100, at 0.5 Hz frequency). The linear viscoelastic region (LVR) where constant G’ and G” values were registered and the beginning of flow at the G’-G’’ crossover strain value was determined. Three interval thixotropy test (3ITT) was used in order to identify the thixotropic behavior of samples. Strain values were set as follows: 0.1% for 5 min (at 0.5 Hz), 100% for 5 min (at 1 Hz) and a last interval of 0.1% for 10 min (at 0.5 Hz) to allow structure recovery. Rheological behavior of nanoemulsions was also studied during rotational shear tests, by increasing the shear rate from 0.1 to 100 s^−1^, and then maintaining it for 3 min at 100 s^−1^ in order to assess the time dependency of samples viscosity. Apparent viscosity was considered as a function of shear rate and time.

##### Color

The optical parameters of the coating forming emulsions were measured with CR410 chroma meter (Konica Minolta, Tokyo, Japan), using C illuminant and observer angle of 2° (Y = 85.9, x = 0.3175, y = 0.3250). The CIELAB L*, a*, and b* values were measured in duplicate for each of the T and S sonicated coating emulsions. The control samples were prepared in the same manner but did not undergo sonication. The difference in color was calculated with Equation (1) [22], where ∆L* = L*_sample_ − L*_control_.

(1) $$\Delta E=(\Delta L^{*2}+\Delta a^{*2}+\Delta b^{*2}{)}^{1/2}$$

The scale proposed by researchers [25] was used for interpreting the results: ΔE = 0–0.5 trace level difference, ΔE = 0.5–1.5 slight difference, ΔE = 1.5–3.0 noticeable difference, ΔE = 3.0–6.0 appreciable difference, ΔE = 6.0–12.0 large difference, and ΔE > 12.0 very obvious difference.

#### 2.2.3. Characterization of Edible Coating Applied to Zucchinis

To characterize the coating material before its application on zucchini, samples of 11 mL of nanoemulsions were cast in silicone trays (diameter 5 cm), dried at room temperature, approximately 22 °C, for 48 h at 50% RH then easily peeled off as membranes [24]. Prior to all investigations, the membranes were preconditioned at 50 ± 3% RH and 25 ± 1 °C, for at least 72 h [26] and further characterized by scanning electron microscopy (SEM) and gas-chromatography.

##### SEM

SEM was used for homogeneity evaluation and thyme essential oil droplets dimensioning. For a better evaluation of the homogeneity, the tested nanoemulsions were cast into edible films, analyzed top-view and cross-section with QUANTA 200 model SEM microscope (former FEI, now Thermo Fisher Scientific, Waltham, MA, USA). Prior to the imagistic evaluation, the samples were attached to the aluminum stub using conductive double-sided carbon adhesive tape. For sectional view, the samples were cut by hand with a razor blade, then sputtered covered with a metallic layer (thickness 7 nm) to obtain conductively surface, using a Sputter Coater system (SPI Supplies, West Chester, PA 19380-4512, USA). Following the coating process, the samples were transferred into the microscope chamber. SEM micrographs (with a wide range between 500× and 5000× magnification) are acquired by detecting the secondary electrons (SE signal) due to the sample surface—primary electron beam interaction and a beam accelerating voltage of 15 kV, under the low vacuum conditions. The analyzed SEM microstructures have a local character, due to the visualized micro-area of about 100 µm^2^.

##### Untargeted Fingerprinting by SMPE Gas Chromatography-Mass Spectrometry (GC-MS)

GC analysis of the coatings’ fingerprints was evaluated in comparison with the volatile profile of thyme essential oil. The volatiles compounds (VOC) from samples were analyzed using a Trace GC-MS Ultra equipment with ionic trap-MS ITQ 900 (Thermo Scientific, Waltham, MA, USA) using an SPME extraction system. The method of incubation, extraction, and analysis was previously optimized. A TG-WAX capillary column (60 m × 0.25 mm, i.d. 0.25 μm) was used while helium (99.996% purity, Messer S.A., Romania) was used as a carrier gas at a flow rate of 1 mL/min.

Samples of 0.750 ± 0.001 g of edible membranes were weight in glass vials (20 mL) with 1 g of saturated (NH_4_)_2_SO_4_ (Redox SRL, Bucharest, Romania) and further spiked with 5 μL 2-octanol 0.651 g/mL (Sigma Aldrich Chemie GmbH, Steinheim, Germany) as internal standard. In the case of thyme essential oil, a sample of 10μL was weighed and the protocol followed the same steps as for the membrane samples. Afterward, the vials were sealed, and the mixture was maintained at 40 °C for 10 min for equilibration before concentration by SPME on a CAR/PDMS fiber.

The extraction of the volatiles under isothermal conditions at 40 °C was made over 20 min followed by 4 min of desorption into the GC injection port. The temperature ramp selected for the analysis was 40 °C isothermal treatment for 4 min followed by an increase to 50 °C at 3 °C/min, with a 1 min holding time, then to 120 °C with 5 °C/min, maintained at 120 °C for 10 min, then up to 170 °C at 4 °C/min and maintained for 10 min and finally to 220 °C at 10 °C/min, when the temperature was kept constant for 10 min. After the final temperature (220 °C), the oven was cooled again to the initial temperature. The temperature of the transfer line in MS was set to 240 °C. Mass spectra were obtained from the full scan of the positive ions resulting from scanning in the 50 to 650 m/z range and operated with an electron impact (EI)-mode of 200 eV. Identification of the volatile compounds was similar to the one described by our research group [24].

#### 2.2.4. Zucchini Coating

The T and S coating nanoemulsions were applied on zucchinis by brushing one time the entire surface of the vegetable with a similar volume of coating solution and draining the excess. The coated zucchinis were maintained for six hours at 22 ± 1 °C until the coating solutions dried. Control samples were maintained uncoated and treated similarly to the coated samples.

#### 2.2.5. Storage of the Coated Zucchinis

Storage of the coated zucchinis was performed in a refrigerated, closed 600 L cabinet, with controlled temperature and humidity (10 ± 1 °C and 75 ± 1%) for 42 days.

#### 2.2.6. Physicochemical Analysis of Coated Zucchinis

The coated zucchinis were analyzed in triplicate during storage, after 1, 5, 12, 16, 21, and 42 day(s).

##### Weight Loss, Water Activity, pH, °Brix of Coated Zucchinis

The weight loss of control and coated zucchinis was determined using randomly selected zucchinis from each sample group with Equation (2):

(2) $$\mathrm{Weight}\;\mathrm{loss}\;\left(\%\right)=\frac{\left(\mathrm{initial}\;\mathrm{weight}-\mathrm{final}\;\mathrm{weight}\right)}{\mathrm{initial}\;\mathrm{weight}}\times 100$$

The water activity of zucchinis’ peel and pulp was evaluated with FA-st lab equipment (GBX, Romans sur Isère, France).

Zucchini peel and pulp were grated, centrifuged and the supernatant was used for pH evaluation with pH meter 315I (WTW, Weilheim, Germany) at 20 ± 1 °C in duplicate on two different zucchini samples. The supernatant was also used for soluble solids (°Brix) evaluation by the Abbe refractometer. Analysis was performed in triplicate and the average values were reported.

##### Texture Evaluation

Zucchinis’ firmness was evaluated at each storage time with Fruit Texture Analyzer (GȔSS, Hamburg, Germany) by measuring the maximum penetration force, F_max_ (N), required for an 8 mm diameter probe to puncture the peel of a coated zucchini sample of 20 × 20 × 20 mm to a depth of 20 mm, at the equatorial part of each sample [27,28]. The test speed was set at 15 mm/s and measured speed at 10 mm/s. All texture measurements were performed in triplicate.

##### Enzyme Extraction

Each day when analyses were scheduled, zucchini samples, coated and uncoated, were peeled with a sharp knife, and further, 10 g of the plant (pulp or peel) were grated. The mash was immediately transferred in 25 mL 0.2 M phosphate buffer pH 6.6 and stored for 45 min in the dark at 4 °C for extraction. Then, the mixture was centrifuged (6000× *g*) at 4 °C. Next, the supernatant was further used as crude enzyme extract for enzymes activity determination. The enzymes activity was determined with a spectrophotometer (UV VIS Cintra 202, Braeside, Australia). All experiments were conducted in triplicate. All the reagents used in the extraction and measurement were of analytic grade or a high degree of purity.

Catalase Activity (CAT) was measured spectrophotometrically with quartz cuvettes [29] as the decrease in absorbance at 240 nm using 2.5 mL of 11 mM hydrogen peroxide and 0.5 mL crude enzymatic extract at 25 °C for 2 min. The blank sample contained 3 mL 11 mM hydrogen peroxide. The CAT was expressed as the change in the absorbance over time, converted in μmol H_2_O_2_/s·kg fresh weight (FW) of the sample analyzed.

Ascorbate peroxidase Activity (APX) was assayed by recording the decrease in absorbance over time of ascorbic acid at 265 nm and 25 °C. The reaction mixture poured in quartz cuvettes was 2.7 mL of 0.1 M phosphate/0.6 mM EDTA buffer, 0.1 mL 5 mM ascorbic acid (Aa), and 0.2 mL of crude extract. The blank sample contained 2.9 mL of 0.2 M phosphate buffer, pH 6.6, and 0.1 mL of 5 mM Aa. The activity was reported in μmol Aa/s·kg FW of the sample analyzed.

Peroxidase Activity (POD) analysis was performed as previously described by [24] with small changes. A reaction mixture of 0.1 mL crude enzymatic extract, 0.2 mL 0.09 M p-phenylenediamine, 0.1 mL of 0.5 M hydrogen peroxide and 2.8 mL of 0.2 M phosphate buffer, pH 6.6 was mixed. The absorbance of the sample was measured against a blank, using a kinetic mode for 2 min at 485 nm and 25 °C. The blank sample contained 0.2 mL 0.09 M p-phenylenediamine, 0.1 mL of 0.5 M hydrogen peroxide and 2.9 mL of 0.2 M phosphate buffer, pH 6.6. The POD activity was expressed in μmol H_2_O_2_/s kg FW.

##### Gas Chromatography for Volatile Fingerprinting in Coated Zucchinis

In the first and last day of storage when analyses were scheduled, zucchini samples, 3.00 ± 0.001 g of zucchini peel coated and pulp were freshly grated and placed in glass vials (20 mL) with 1 g of saturated (NH_4_)_2_SO_4_ (Redox SRL, Bucharest, Romania) further spiked with 5 μL 2-octanol 0.651 g/mL (Sigma Aldrich Chemie GmbH, Steinheim, Germany) as internal standard. Then analysis was performed as described at Section 2.2.3.

##### Color Changes of the Coated Zucchinis

Color variations of the coated zucchinis during the 42 days of storage were analyzed with MiniScan XE Plus colorimeter (Hunter Associates Laboratory, Inc., Reston, VA, USA) with D65 illuminant and an observation angle set at 10°, calibrated with standard white and black tiles (X = 80.25, Y = 85.03, Z = 90.35). The CIELAB L*, a*, and b* values were measured in three different points of peel and pulp of two zucchinis for each analysis day. The difference in color ΔE was estimated with Equation (1), considering $\Delta L^{*}{=L}_{\mathrm{coated}}^{*}-L_{\mathrm{uncoated}}^{*}$. The same scale as described in Section 2.2.2 was used for interpreting the difference in color results.

##### Sensory Evaluation of Coated Zucchinis

A panel of seven trained evaluators applied a 9 point hedonic scale adapted from [1] for sensorial attributes (appearance—peel and pulp color, defects determined by manipulation and storage or by molds, firmness, odor) and for overall quality based on color, texture, and odor (9: excellent; 5: acceptable limit of marketability; 1: extremely poor) to quantitatively assess coated zucchinis compared to uncoated ones used as control. Two samples of each type of coating were presented for sensory analysis.

#### 2.2.7. Microscopic Evaluation of Coatings Applied to Zucchinis

Evaluation of the zucchinis’ coated surface during the 42 days of storage was performed with Leica EY4D (Leica, Wetzlar, Germany) stereomicroscope.

#### 2.2.8. Statistical Analysis

Data are presented as mean ± standard deviation (SD). The significance of the factors was statistically analyzed with Minitab18 software (Coventry, UK) using the analysis of variance (ANOVA) and Tukey’s post-hoc test enabled the evaluation of significant differences among groups (*p* < 0.05).

## 3. Results and Discussions

### 3.1. Characterization of Coating Nanoemulsion

#### 3.1.1. Rheometric Measurements

Rheological properties of nanoemulsions were reported to differ from conventional ones due to smaller droplet sizes [30].

**Figure 1:**
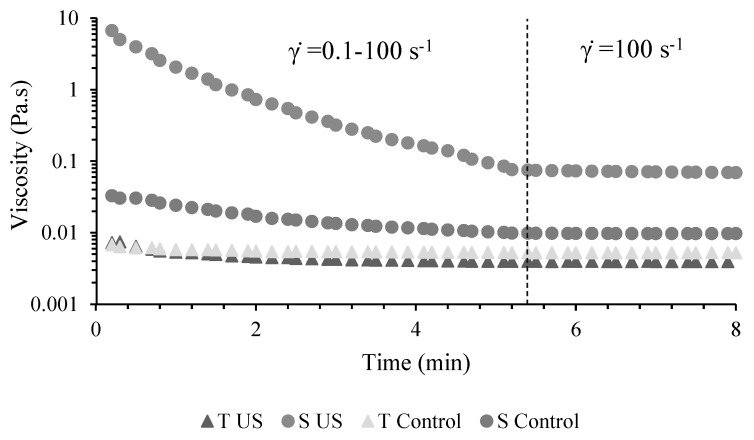
Rheological behavior during rotational flow test of obtained whey protein-thyme EO nanoemulsions. T US-ultrasonicated T nanoemulsion, S US-ultrasonicated S nanoemulsion, T Control, S Control.

The rheological behavior of whey protein-thyme EO nanoemulsions obtained in the present study, during rotational shear tests, is depicted in Figure 1. All samples presented a shear-thinning behavior when ramping the shear rate from 0.1 to 100 s^−1^. The phenomenon was more accentuated for S nanoemulsions in comparison to T samples, due to the significantly higher (*p* < 0.05) initial viscosity values of the first. Other researchers [31] also used S-based formulae containing guar gum in whey protein isolate nanoemulsions in order to increase its viscosity. The authors stated viscosity values of 28–51 mPa.s when ramping the shear rate from 0.1 to 1000 s^−1^.

In the second part of the test, when maintaining shear rate values constant, a time-independent pseudoplastic behavior could be observed for all four samples. The average viscosity value of ultrasonicated S nanoemulsion (S US) in the constant shear rate domain was significantly higher from the other three samples (*p* < 0.05), while the S Control sample registered similar viscosity to both T samples (T US and T Control) containing nanoemulsions (Table S1).

**Figure 2:**
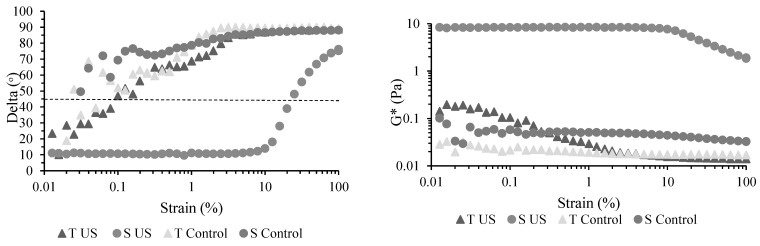
Rheological behavior during small-amplitude oscillatory strain sweep test of the whey protein-thyme EO nanoemulsions. T US—sonicated T nanoemulsion, S US—sonicated S nanoemulsion, T Control—nanoemulsion, S Control—nanoemulsion.

Viscoelastic characteristics of obtained nanoemulsions in terms of phase shift (*δ*°) and G* evolution as a function of applied strain are presented in Figure 2 on a semi-logarithmic scale. Phase shift *δ* is a measure of the sample’s viscoelasticity. For a viscoelastic solid-like behavior, delta values <45° are registered, while values >45° will be characteristic of viscous liquid-like structures [32]. The onset of flow corresponding to the G′-G″ intersection is registered at 45°, while 90° is characteristic of Newtonian behavior. G* is a measure of the deformation of the sample and embraces both elastic (G′) and viscous moduli (G″). The results presented in Figure 2 show that only S US nanoemulsions presented an LVR—the structure being resistant to the applied strain up to a value of 10%. At this point, particles start moving, however, the flow was not yet initiated up to a strain of 25% (Table S1). Additionally, S US nanoemulsions presented the highest consistency (G* values) in comparison to the other three samples (*p* < 0.05). T samples registered a very weak liquid-like structure with no significant effect resulting after sonication (*p* > 0.05). Similar viscous behavior for the entire strain domain tested (Table S1) was registered for S nanoemulsion with no sonication (S Control).

Given the rheological behavior determined during the strain sweep test, the only appropriate sample for the 3ITT was the S US nanoemulsion (Figure S1). Three interval thixotropy test (3ITT) simulates structural breakdown during the coating process (second interval) together with structural regeneration at rest. A complete time-dependent recovery of the initial state upon reduction of the load will indicate a thixotropic behavior (i.e., a high-quality coating). S US sample showed a distinct behavior with a complete restructuring at rest. A predominant viscous behavior, as expected, could be observed for both T samples and control S (Figure S1).

#### 3.1.2. Color Estimation

The nanoemulsions used for coating the zucchinis were assessed from the point of view of the color parameters L*–luminosity (lower values representing darker colors and vice versa, maximum 100), a*–Chroma range green (negative values)/red (positive values) and b*–Chroma range blue (negative values)/yellow (positive values). The total color difference ΔE* was estimated by using control samples with the same composition, which did not undergo sonication.

Results presented in Table 1 show that the luminosity of both T and S nanoemulsions are significantly lower than those of the control emulsions; b* value for T nanoemulsion is significantly higher than that of the T Control emulsion which indicates a more pronounced yellow tone for T nanoemulsion. Similar changes related to the addition of EOs and/or T to emulsions and to the sonication step were reported by other research groups [33,34,35].

According to the classification proposed by researchers [25] both T (ΔE* 2.33 ± 0.40) and S (ΔE* 2.45 ± 0.28) emulsions exhibited a noticeable difference in color compared to the control samples, which did not undergo sonication. The explanation resides in the fact that the size of thyme EO and glycerol droplets are greatly reduced during sonication, which triggers a weaker light scattering [22] compared to that of the bigger droplets of the control emulsions.

### 3.2. Characterization of Membranes with T and S Nanoemulsions

#### 3.2.1. SEM Coating Evaluation

**Figure 3:**
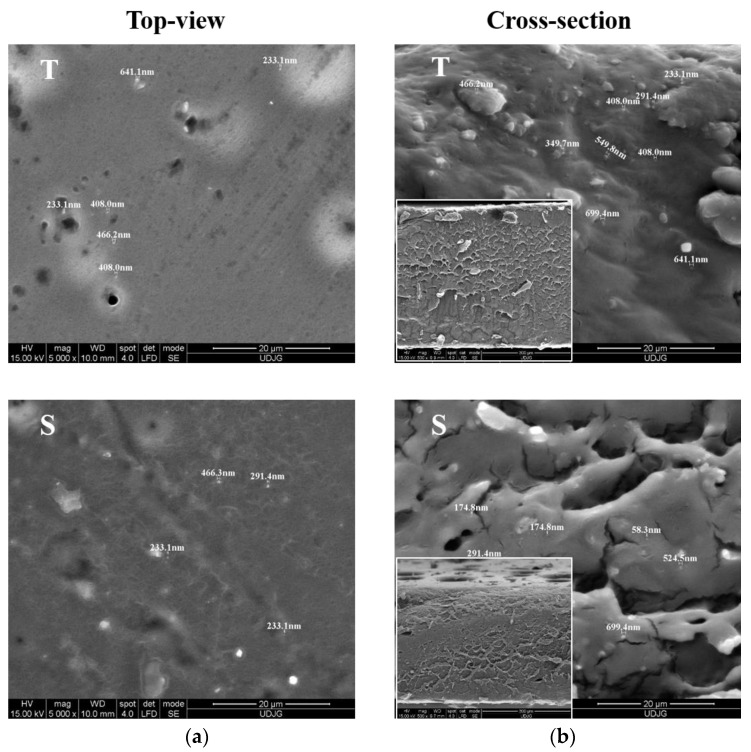
SEM micrographs (20 µm scale bar) of: (**a**) top-view; and (**b**) cross-section presenting the spherical morphology and uniform size distribution of thyme EOs with T & S nanoemulsions.

SEM microstructures are presented for membranes that were later used for zucchini coating. As shown in Figure 3, the shape, size, and distribution of thyme oil droplets prepared using two stabilizers, T and S, were observed by using the SEM technique. The presence of oil nanoparticles in the film is obvious, and they appear as light spots in the darker film matrix. The oil particles are uniformly distributed in the emulsion, in all scanned micro-areas. Top-view images (Figure 3a) taken on both types of nanoemulsions reveal a few numbers of oil droplets, having spherical morphology [36], whereas the cross-section images (Figure 3b) show more clearly and a higher density of nanoparticles incorporated into the film. SEM images indicate some open microporosity throughout the T-membrane which were previously reported by similar studies [37] (Figure 3a). The discontinuity in the structure could be due to the hydrophobic character of the essential thyme EO [38]. T-nanoemulsions showed bigger particles (mean size around 450 nm), and slight aggregation can be observed. In addition to the thyme oil droplets, some glycerol droplets as darker circular structures can be seen on the surfaces [39]. On the other hand, S membranes have a reduced porosity, and their structure looks more compact, integer (Figure 3b). As it can be observed, the S membrane is more stable and contains fine particles (mean diameter of particles is reduced around 380 nm, measured in transversal section). Although there is no consensus definition on the critical size values of the nanoparticles, being considered either 100 nm, 200 nm, or 500 nm [40], due to the above-reported mean diameter value and spheroid shape of most of the oil droplets viewed in this study, we have considered them nanoparticles in agreement with other researchers [41].

Therefore, we can consider both T and S increase the thyme EO particles dispersion throughout the film matrix, showing their uniform size distribution. A decrease in the mean size of the oil droplets might suggest S for coatings with superior properties.

#### 3.2.2. GC Fingerprint

The thyme EO used in this study is characterized by the following most abundant and representative compounds: 4-carene, caryophyllene, cis-geraniol, cis pinen 3-ol, menthatriene, cyclooctene, ocimene, and thymol. More than 90% of the VOCs fingerprint in thyme EO and in the T and S-membranes, consisting of 35 components, were tentatively identified with Nist 08 library and indicated in Table 2.

These results are different from the ones reported by [42] where the most abundant component was γ-terpinene, while here, 4-carene had the highest concentration. Other *Thyme* genera revealed that the most abundant components of the extracted essential oils from *T. algeriensis* [43] were α-pinene, 1,8 cineol, camphor, and caryophyllene oxide, while in *T. vulgaris,* researchers indicated [44] that p-cymene, γ-terpinene, and thymol had the highest concentrations. Besides plant genus, the difference in composition could have been influenced by other biological factors (phenological stage, age of plant, parts of plant used in extraction), growth environment, and by technological factors (drying, storage conditions, extraction conditions, and parameters) [42].

The heat map in the last column of Table 2 shows the differences in VOCs peak area of S and T membranes, indicating in green the most abundant components in S-formula like 4-carene and caryophyllene while in reading the most abundant components in the T formula were α-pinene, camphene, and cis-geraniol. The total area of VOCs in the S matrix was 33% higher than in the T membrane, thus it was considered more prone to retain volatile. However, valuable biologically active terpenes such α-pinene and camphene were present in lower concentrations in the S membrane compared to the T membrane.

### 3.3. Physicochemical Analysis of the Coated Zucchinis

#### 3.3.1. Weight Loss, Water Activity, pH, Brix

Zucchini is a highly perishable vegetable that rapidly reduces its quality during postharvest storage, mostly due to considerable water loss [45]. The coating formulae presented in this study were optimized in previous research [17] and care should be taken when adding essential oils in coatings applied on plants not to exceed the limit that could produce prooxidant effects (Figure S2-Supplementary file).

**Figure 4:**
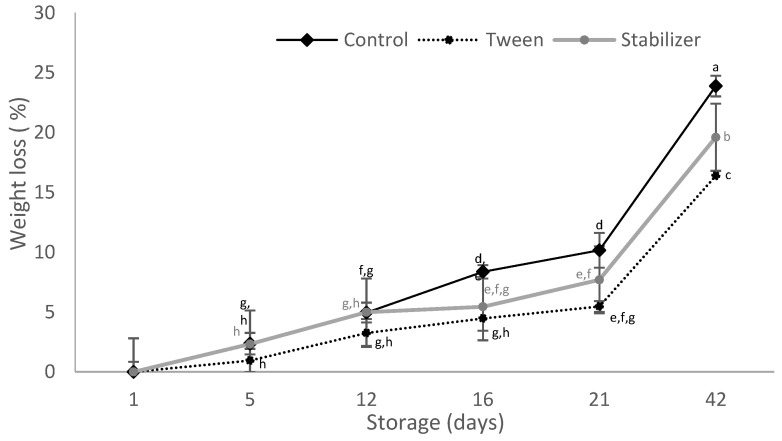
Weight loss of zucchinis during 42 days of storage. The data are mean ± SD of three replicates from two zucchinis. Different letters indicate significant differences with ANOVA and Tuckey post-hoc test at *p* < 0.05.

The weight loss of uncoated zucchinis evaluated in this study increased with storage time (Figure 4); similar behavior was reported during the usual two weeks of storage reported by other studies [3,5].

Zucchinis S coated had the same weight loss as the uncoated ones during the first 12 days of storage. The weight loss was significantly low (*p* < 0.05) and prevented by S coating during the last 26 days of storage, leading to 85% lower weight loss compared to the control in the 42 days of storage. T triggered the smallest weight loss of all analyzed samples during the entire storage period, leading to 66% weight loss compared to uncoated zucchinis, after 42 days of storage. These results indicate that T coating had the best performance in zucchini during the entire 42 days of storage.

Previous research [46] indicated that shrivel symptoms on yellow crookneck summer squash became apparent following 18% weight loss and were moderate by 24% weight loss. This water loss limit at which these cucurbits become unmarketable or have to be sold for a lower price is also important in terms of losses of appearance, texture, and nutritional quality [11].

T-coated zucchinis reached 16.48 ± 0.45 weight loss (%) and S-coated ones reached 21.16 ± 1.11 weight loss (%) after 42 days of storage.

On the first day of storage, the water activity in zucchinis’ peel was 0.960 ± 0.02 and in the pulp 0.975 ± 0.01, pH_peel_ 6.00 ± 0.25, pH_pulp_ 5.80 ± 0.13, which are similar to other reported values [31,47].

The soluble solids (°Brix) of zucchinis were 5.74 ± 0.3 at the beginning of storage, similar to other reported values [6,48]. After 42 days of storage, the control had the lowest value of the analyzed samples, of 4.6 ± 0.2° Brix, S-coated zucchinis 5.2 ± 0.7° Brix, while T-coated zucchinis had 5.85 ± 0.4° Brix.

#### 3.3.2. Texture Analysis

Firmness is an important quality attribute of raw vegetables, directly correlated with their marketability [49]. Considering vegetables’ texture dependency on the rate and extent of water loss leading to softening of cellular tissues [50], this study evaluated the effect of coating nanoemulsions on the texture of zucchinis during 42 days of storage.

**Figure 5:**
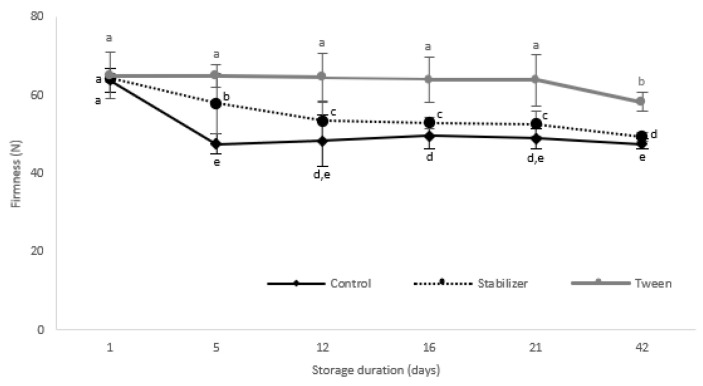
Firmness of coated zucchinis during storage. The data are mean ± SD of three replicates from two zucchinis. Different letters indicate significant differences with ANOVA and Tuckey post-hoc test at *p* < 0.05.

As shown in Figure 5, firmness significantly declines in control samples in the first 5 days of storage (*p* < 0.05), which is similar to other reported values [3]. T-coated zucchinis did not exhibit significant texture changes compared to the initial state until after the 21st day. The comparison between the beginning and the end of storage shows a 25.73% firmness decrease for control (uncoated zucchini), a 23.30% decrease for the S-coated ones, while the T-coated ones had the lowest firmness decrease, of only 10.38% in 42 days of storage. These results indicate T coating as being more effective in delaying the softening of zucchinis during prolonged storage.

#### 3.3.3. Enzymatic Activity

Zucchini storage can result in exceeding ROS production by the respiratory chain [51,52] and, if unbalanced by the antioxidant system, it generates alteration of the cell wall metabolism, the breakdown of the fatty acid in membrane lipids, and finally produce fruit decay. Enzymes such as catalase (CAT) and ascorbate peroxidase (APX) are part of the ROS scavenging systems and they are activated upon postharvest storage, having the ability to remove/neutralize intermediates of monovalent oxygen reduction [51,53].

To evaluate the potential protective effect of coatings formulae applied on zucchini, the catalase (CAT), peroxidase (POD), and ascorbate peroxidase (APX) were measured during storage at 10 °C, in pulp and peel.

**Figure 6:**
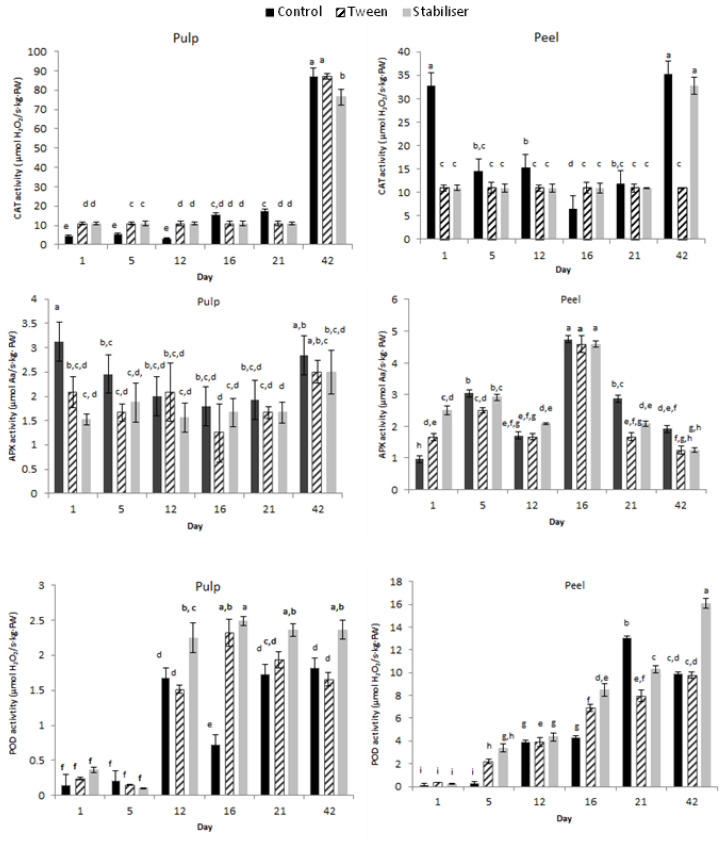
Catalase (CAT), ascorbate peroxidase (APX), and peroxidase (POD) activity in coated zucchini pulp and peel during six weeks of storage. The data are mean ± SD of three replicates from two zucchinis. Different letters indicate significant differences with ANOVA and Tuckey post-hoc test at *p* < 0.05.

Catalase activity was almost constant in pulp (*p* > 0.05) and peel (*p* > 0.05) of the coated zucchinis for 21 days. It can be noticed that after coating the peel presented a 2.75-fold lower CAT activity compared to control. The same decrease was reported by [54] in preconditioned zucchini at 15 °C followed by storage at 5 °C. However, this research reported on the 42nd day of storage a significant increase (*p* < 0.05) in CAT activity, of almost 3-fold in peel sample containing S, and more than 9-fold in pulp for both zucchini samples coated with T and S (Figure 6).

The APX markedly increased in the exocarp on the 16th day of storage in both S and T samples (*p* < 0.05) followed by a decrease on the 42nd day to values lower than on the first day (*p* < 0.05). The same tendency to increase in AOX during storage was reported by [55] on the 9th day of storage and it is most probably related to the increase in ROS compounds. In pulp, the APX activity remained lower than control in both S and T samples for all the storage period, though on the 42nd day of storage a significant increase (*p* < 0.05) was observed in both S and T samples (Figure 6).

The POD content constantly increased in peel during storage, the highest activity being registered for the S sample at the end of storage. The same trend was reported by [51] in zucchini peel kept at 10 °C, with values considerably higher than in control and consistent with the reported results of [56].

In comparison with the control sample, POD activity increased in pulp for the T and S samples from the 12th day onward, however, the increase was considerably more reduced than in peel. Significant differences (*p* < 0.05) were registered for the zucchini coated with S and T nanoemulsions at the end of storage in both peel and pulp with the highest POD activity for the S sample.

#### 3.3.4. VOCs Profile Changes in Stored Coated Zucchinis Assessed by SPME GC-MS

**Figure 7:**
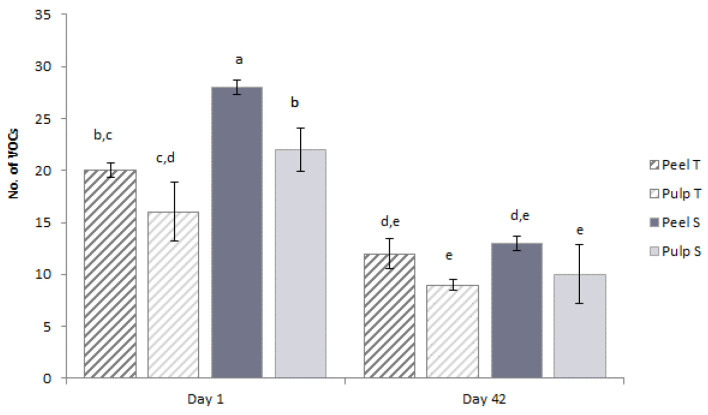
The dynamics of VOCs in T and S coated zucchinis’ peel and pulp during storage. Different letters indicate significant differences with ANOVA and Tuckey post-hoc test at *p* < 0.05.

The untarget fingerprint of the zucchinis’ peel and pulp during the entire storage time highlighted that volatiles, such as α-pinene, guaiene, and caryophyllene, from the outer coating are migrating in the pulp. As shown in Figure 7, on the first day of storage, the S coating had a significantly higher number of VOCs in peel (*p* < 0.05) compared with the T, while after 42 days of storage time, the total number of VOCs was smaller than in the first day but similar in peel in both formulae.

The significantly reduced (*p* < 0.05) concentration in VOCs in the 42nd day of storage compared with the first day could be explained by the depletion of terpenes with the high volatility that left the coating to the environment until equilibrium was reached. In pulp we could find a high number of volatiles after the first day of storage and with 37.5% (*p* < 0.05) more in the S-coating compared to T. On the 42nd day of storage, the lower concentration in VOCs found in pulp compared with the beginning of storage was similar in the coating with T and S (*p* > 0.05).

#### 3.3.5. Color Changes of the Coated Zucchinis during Storage

All color parameters for uncoated zucchinis were similar to other values reported in studies using the same variety of zucchini [57]. The peel of zucchinis (Table 3) coated with T nanoemulsion exhibited a significant decrease of luminosity values over the 42 days of storage, corresponding to lighter tones at the end of storage (L_day1_ = 58.44 ± 2.44 vs. L_day42_ = 79.22 ± 6.75). The negative values for a* parameter of all zucchini peals indicate the green tone of the samples; assessment of a* and b* values in the first day shows that neither one of the coatings significantly changed the green-yellow appearance of the zucchinis over the 42 days of storage. Based on [25] classification the difference in color just after the coating was appreciable for T coated zucchini and large difference for the S coated zucchinis, compared to the control uncoated zucchini.

The values for luminosity L* in pulp samples of T and S coated zucchinis vs. uncoated ones indicate that both coated zucchinis were significantly lighter at the end of storage (Table 4). The negative a* values for pulp are not significantly different during the entire storage duration across all samples, which shows that the outside coating did not negatively affect the color of the zucchini endocarp or its respiratory capacity. The pulp difference in color is not significantly different in the first 16 days of storage, which is in accordance with the usual shelf life of zucchini of approximately 14 days [58]. After 42 days of storage, the T-coated zucchinis are noticeably different from the control (ΔE* = 1.96 ± 0.01), while S-coated ones exhibit a large difference in color compared to the control (ΔE* = 11.11 ± 1.67), which can be easily observed in Table 4.

**Figure 8:**
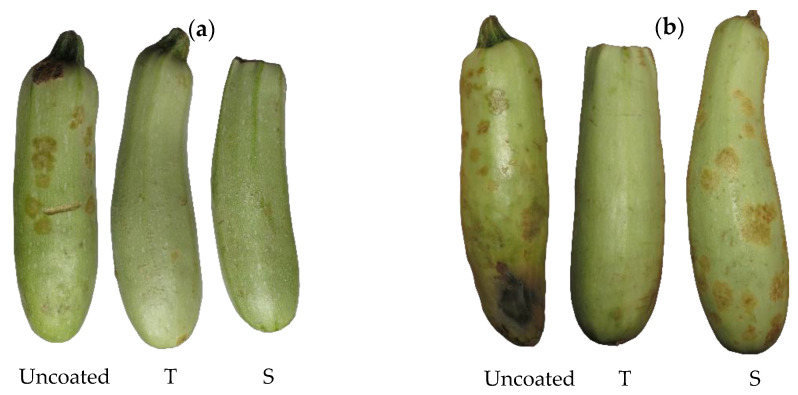
Zucchinis after 12 (**a**) and 42 (**b**) days of storage.

Figure 8 presents the analyzed zucchinis after 12 and 42 days of storage.

Moderate shrivel appearance was visible only for S-coated zucchinis after 42 days of storage, which correlates with 21.16 ± 1.11 weight loss (%) and the results reported by other researchers [45].

#### 3.3.6. Sensory Evaluation of Coated Zucchinis

**Figure 9:**
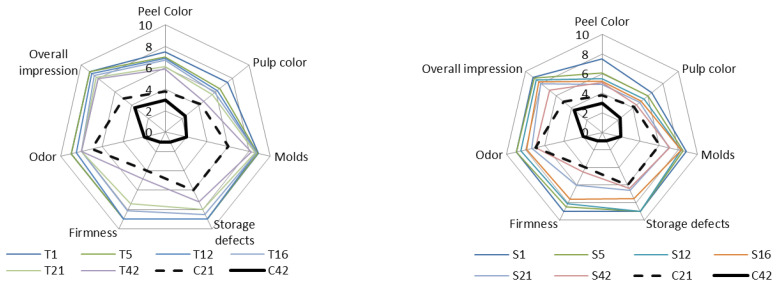
Influence of the coating on zucchini sensory evaluation after 42 days of storage. T1, T5, T12, T16, T21, and T42 represent the average score for each individual sensory attribute obtained by the coated zucchini with Tween 20 stabilizer in the 1st, 5th, 12th, 16th, and 42nd day of storage, respectively; S1, S5, S12, S16, S21, S42 represent the average score for each individual sensory attribute obtained by the coated zucchini with gums mixture as a stabilizer in the 1st, 5th, 12, 16th, and 42nd day of storage, respectively; C21 and C42 represent the average score for each individual sensory attribute obtained for uncoated zucchini (control) in the 21st and 42nd day, respectively.

Figure 9 presents the acceptance scores for coated zucchinis. All quality parameters decreased during storage for all samples, although S-coated zucchinis showed a quicker deterioration compared to T-coated ones during the entire period of storage. Control samples had lower scores compared to both T and S coated zucchinis; after 21 storage days, the uncoated zucchinis obtained scores below 5 which corresponded to an unmarketable state and their shelf-life end.

The controls’ low scores were due to peel and pulp color, off-odor, and overall impression. The T-coated zucchinis obtained the highest scores of all samples, which were higher than the marketable acceptability up until the 21st day. T coating ensured significantly (*p* < 0.05) higher scores compared to uncoated samples, is especially appreciated by the panelists for their peel color, lack of molds or storage defects, firmness, and natural-like odor, with an overall impression of 8.2 ± 0.6 out of a maximum of 9. Panelists’ opinion indicated that the T coated zucchinis could have an extended shelf life between 21–42 days, which corresponds to 50–200% shelf-life extension. S coatings had a much lower protective effect against storage deterioration from the beginning of the study, with higher intensity of degradation processes taking place during storage. S coated zucchinis reached a maximum marketability period of only 16 days compared to a usual two weeks for control zucchinis, mostly due to peel &pulp color, molding, loss of firmness, and off-odors.

#### 3.3.7. Stereomicroscopic Evaluation of Coating Applied to Zucchinis

**Figure 10:**
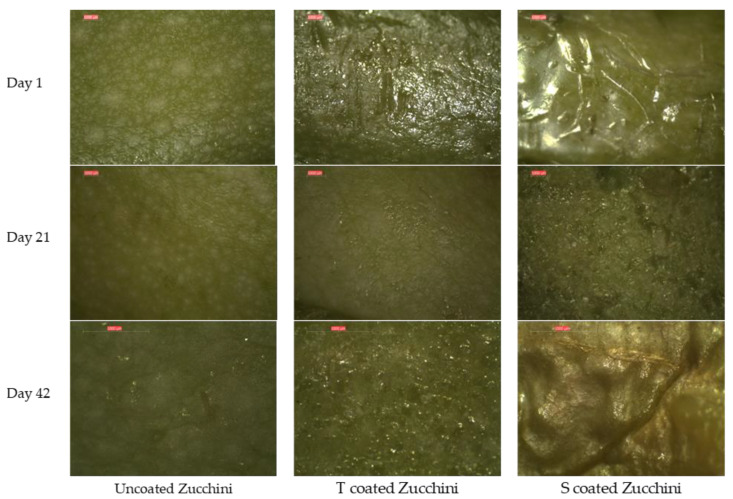
Zucchini peel magnified by stereomicroscope.

Figure 10 shows that at the beginning of storage the peel of all zucchinis is smooth, the S coating is visible and shinier compared to the T coating. As storage evolves, the physiological loss of water is visible in the uncoated sample after 21 days and less visible in the coated zucchinis. While the T coating is smooth and ensured a delay of senescence appearance, the S coating exhibits surface cracks, which are most probably related to the observed shriveling of the zucchini. After 42 days of storage, the control zucchini has a dull color, the S-coated one presented accelerated senescence, while the T coating enabled maintenance of firm texture and vibrant, natural color, which is in accordance with the situation depicted in Figure 8.

## 4. Conclusions

This study shows that ultrasound treatment at 35% amplitude for 3 min ensures the formation of thyme essential oil nanoemulsions with whey protein stabilized by a mix of guar and arabic gum (S) or by polysorbate (T).

The nanoemulsions with S displayed higher consistency than the control and samples with T, while the nanoemulsions with T could not be rheologically differentiated from control due to their weak viscosity. The SEM indicated that the S coating is able to form more compact structures and with smaller oil drops than T. The different capacity of retaining volatiles of the T and S coatings evidenced by GC fingerprinting also reflects the preservative capacity displayed by the coatings. Several volatiles, such as α-pinene, guaiene, and caryophyllene, are quickly migrating from the outer layer in the pulp from the first day of storage. At the end of storage zucchinis’ VOC profile in peel and pulp is significantly reduced.

While rheology, SEM, and GC indicated that S coating is superior to T coating, when applied on zucchini, lower water loss, higher firmness, better sensorial profile, and better peel color were ensured by T coating. These findings suggest that, when applied on zucchini, T coating is more efficient to act as a dynamic membrane allowing plant breathing and reducing ROS occurrence in the peel, a fact demonstrated by the significantly less POD activity in peel at the end of storage compared to S coating. Additionally, the T coating has the potential of increasing zucchinis’ shelf life between 21–42 days, corresponding to 50–200% shelf-life extension.

## Figures and Tables

**Table 1:** Color parameters for nanoemulsions.

	Parameters	L*	a*	b*	ΔE*
Sample					
T Control		73.23 ± 0.07 **^A^	−1.42 ± 0.01 ^A^	4.37 ± 0.02 ^A^	
T		71.06 ± 0.32 ^B^	−1.07 ± 0.31 ^A^	5.15 ± 0.66 ^B^	2.33 ± 0.40 ^A^
S Control		71.75 ± 0.07 ^C^	−1.48 ± 0.01 ^A,B^	5.06 ± 0.00 ^B^	
S		70.29 ± 0.19 ^D^	−1.88 ± 0.14 ^B^	7.05 ± 0.24 ^B^	2.45 ± 0.28 ^A^

** The data are mean ± SD of three replicates from two reading. Different letters indicate significant differences with ANOVA and Tuckey post-hoc test at *p* < 0.05.

**Table 2:** Percentage composition of the volatile compounds (VOCs) of thyme EO and their presence in T and S membranes.

No	KI	Ions	Compound	Formulae	Thyme EO (%)	T (%)	S (%)	Heatmap Area (S-T)
1	843	91;93;77;51;121	3-Thujene *^,a^	C_10_H_16_	1.21 ± 0.05 ^a,E,F^	0.21 ± 0.05 ^c,E,F^	0.54 ± 0.02 ^b,D^	
2	890	81;107;135;150;53	Tricyclene	C_10_H_16_	1.63 ± 0.08 ^a,E,F^	0.14 ± 0.01 ^c,F^	0.86 ± 0.06 ^b,D^	
3	922	91;93;77;51;121	ç-Terpinene	C_10_H_16_	0.59 ± 0.04 ^a,E,F^	0.28 ± 0.02 ^c,E,F^	0.42 ± 0.02 ^b,D^	
4	966	91;93;77;65;105	α-Phellandrene	C_10_H_16_	0.44 ± 0.03 ^aF^	0.08 ± 0.03 ^c,F^	0.25 ± 0.02 ^b,D^	
5	986	119;91;115;135;134	1,3,8-Menthatriene	C_10_H_14_	1.25 ± 0.12 ^b,E,F^	0.51 ± 0.02 ^c,E,F^	1.63 ± 0.13 ^a,D^	
6	992	91;93;77;121;136	2-Bornene	C_6_H_10_	0.86 ± 0.07 ^a,E,F^	0.21 ± 0.02 ^c,E,F^	0.60 ± 0.05 ^b,D^	
7	1024	67;93;91;108;65	Camphenone	C_5_H_8_	0.03 ± 0.00 ^c,F^	0.37 ± 0.02 ^a,E,F^	0.24 ± 0.02 ^b,D^	
9	1082	91;115;117;132;77	1H-Indene	C_10_H_12_	0.03 ± 0.00 ^a,F^	0.02 ± 0.00 ^b,F^	0.03 ± 0.00 ^a,D^	
10	1096	91;93;77;121;111	α-Pinene	C_10_H_16_	2.79 ± 0.03 ^a,E^	1.72 ± 0.12 ^b,E,F^	1.11 ± 0.01 ^c,D^	
11	1107	105;119;91;120;77	Aromadendrene	C_15_H_24_	0.01 ± 0.00 ^a,F^	0.04 ± 0.00 ^b,F^	0.01 ± 0.00 ^a,D^	
12	1119	95;67;108;93;81	Limonene	C_10_H_16_	1.15 ± 0.01 ^c,E,F^	0.34 ± 0.02 ^a,E,F^	0.23 ± 0.01 ^b,D^	
13	1127	91;77;93;67;121	Camphene	C_10_H_16_	1.91 ± 0.12 ^a,E,F^	1.63 ± 0.15 ^a,E,F^	1.21 ± 0.11 ^b,D^	
14	1132	91;77;93;121;107	Santolina triene	C_10_H_16_	0.62 ± 0.02 ^a,E,F^	0.43 ± 0.03 ^b,E,F^	0.56 ± 0.05 ^a,D^	
15	1143	93;91;95;67;121	1,3,6-Heptatriene	C_7_H_10_	0.98± 0.05 ^a,E,F^	0.64 ± 0.05 ^b,E,F^	0.96 ± 0.06 ^a,D^	
16	1150	91;105;77;119;133	Caryophyllene	C_15_H_24_	22.03 ± 1.58 ^a,B^	9.46 ± 1.02 ^c,C^	14.52 ±0.15 ^b,B^	
17	1176	91;77;93;105;119	Seychellene	C_15_H_24_	0.48 ± 0.05 ^a,F^	0.48 ± 0.02 ^a,E,F^	0.40 ± 0.01 ^b,D^	
18	1188	91;93;67;77;121	Ocimene	C_10_H_16_	0.24 ± 0.00 ^b,F^	0.23 ± 0.00 ^a,F^	0.24 ± 0.00 ^b,D^	
19	1189	105;161;91;119;133	Germacrene	C_15_H_24_	0.36 ± 0.03 ^a,F^	0.11 ± 0.01 ^c,F^	0.24 ± 0.01 ^b,D^	
20	1193	91;93;77;79;67	cis-α-Bisabolene	C_15_H_24_	0.41 ± 0.03 ^a,F^	0.40 ± 0.03 ^a,E,F^	0.46 ± 0.03 ^a,D^	
21	1221	93;91;121;67;136	4-Carene	C_10_H_16_	30.77 ± 3.21 ^b,A^	40.03 ± 2.75 ^a,A^	41.42 ± 3.59 ^a,A^	
22	1228	91;77;93;119;65	Cis pinen 3-ol	C_10_H_16_O	5.32 ± 0.22 ^b,D^	5.20 ± 0.34 ^b,D^	6.74 ± 0.58 ^a,C^	
23	1299	91;93;79;67;121	3-Carene	C_10_H_16_	0.72 ± 0.01 ^a,E,F^	0.84 ± 0.06 ^a,E,F^	0.84 ± 0.07 ^a,D^	
24	1313	91;93;67;121;123	cis-Geraniol	C_10_H_18_O	10.64 ± 1.58 ^b,C^	17.05 ± 1.33 ^a,B^	13.58 ± 1.12 ^b,B^	
25	1330	91;77;119;134;51	cis-Cyclooctene	C_8_H_14_	0.50 ± 0.01 ^c,E,F^	1.97 ± 0.15 ^a,F^	1.65 ± 0.15 ^b,D^	
26	1334	91;93;67;121;65	α-Ocimene	C_10_H_16_	0.25 ± 0.01 ^c,F^	0.38 ± 0.01 ^a,E,F^	0.34 ± 0.02 ^b,D^	
27	1346	91;77;119;65;51	trans-Cyclooctene	C_8_H_14_	0.06 ± 0.00 ^a,F^	0.09 ± 0.01 ^a,E^	0.08 ± 0.04 ^a,D^	
28	1382	91;77;105;107;67	Lanceol-cis	C_15_H_24_O	0.02 ± 0.00 ^a,F^	0.05 ± 0.00 ^b,F^	0.02 ± 0.00 ^a,D^	
29	1387	91;79;77;105;57	Ledene oxide	C_15_H_24_O	0.29 ± 0.01 ^c,F^	0.62 ± 0.03 ^a,E,F^	0.38 ± 0.02 ^b,D^	
30	1411	91;105;107;67;119	Azulene	C_10_H_8_	0.01 ± 0.00 ^a,F^	0.07 ± 0.00 ^b,F^	0.03 ± 0.00 ^c,D^	
31	1415	79;77;67;91;93	Myrtenol	C_10_H_16_O	0.02 ± 0.00 ^a,F^	0.08 ± 0.00 ^c,F^	0.05 ± 0.00 ^b,D^	
32	1439	91;105;119;79;161	α-Guaiene	C_15_H_24_	0.01 ± 0.00 ^b,F^	0.02 ± 0.00 ^a,F^	0.02 ± 0.00 ^a,D^	
33	1455	91;105;119;77;131	Spathulenol	C_15_H_24_O	0.01 ± 0.00 ^a,F^	0.14 ± 0.01 ^b,F^	0.03 ± 0.00 ^b,D^	
34	1473	135;115;91;150;79	Thymol	C_10_H_14_O	0.04 ± 0.01 ^b,F^	0.11 ± 0.05 ^a,F^	0.08 ± 0.02 ^a,D^	
35	1485	91;119;77;136;67	2,5-Octadecadienoic acid	C_19_H_34_O_2_	0.02 ± 0.00 ^c,F^	0.1 ± 0.01 ^a,F^	0.06 ± 0.01 ^b,D^	

* different small caps letters indicate significant differences (*p* < 0.05) on rows and capital letter on columns, estimated by post-hoc Tuckey test; KI-Kovats Index; ^a^ Data reported are mean ± SD of three replicates.

**Table 3:** Color evaluation of zucchini peel during 42 days of storage.

Storage	Sample	L*	a*	b*	ΔE*
Day 1	Uncoated	62.55 ± 0.23 ^B,C,D,E,F^	−7.28 ± 0.07 ^E^	27.39 ± 0.33 ^C,D,E,F,G^	
	T	58.44 ± 2.44 ^C,D,E,F,G,H^	−7.24 ± 0.27 ^E^	29.51 ± 1.58 ^A,B,C,D,E,F,G^	4.62 ± 2.88 ^B,C^
	S	51.30 ± 0.26 ^H^	−7.15 ± 0.02 ^D,E^	28.11 ± 2.98 ^C,D,E,F,G^	11.28 ± 0.48 ^A,B,C^
Day 5	Uncoated	63.70 ± 0.10 ^B,C,D,E^	−6.97 ± 0.17 ^C,D,E^	26.58 ± 0.43 ^D,E,F,G^	
	T	52.67 ± 7.23 ^F,G,H^	−5.23 ± 0.88 ^B,C^	24.57 ± 2.68 ^F,G^	11.34 ± 5.86 ^A,B,C^
	S	57.91 ± 1.21 ^C,D,E,F,G,H^	−6.09 ± 0.86 ^C,D,E^	30.39 ± 0.18 ^A,B,C,D,E,F^	6.99 ± 1.00 ^A,B,C^
Day 12	Uncoated	64.43 ± 0.08 ^B,C,D,E^	−7.07 ± 0.07 ^D,E^	32.96 ± 0.20 ^A,B,C^	
	T	58.66 ± 0.01 ^B,C,D,E,F,G,H^	−5.90 ± 0.34 ^C,D,E^	27.27 ± 1.46 ^C,D,E,F,G^	8.19 ± 0.44 ^A,B,C^
	S	51.90 ± 4.31 ^G,H^	−5.47 ± 0.44 ^C,D^	23.80 ± 3.57 ^G^	15.61 ± 5.59 ^A^
Day 16	Uncoated	61.90 ± 0.16 * ^B,C,D,E,F,G^	−7.03 ± 0.03 ^D,E^	29.61 ± 0.12 ^A,B,C,D,E,F,G^	
	T	63.42 ± 1.68 ^B,C,D,E^	−5.63 ± 0.96 ^C,D,E^	30.25 ± 2.75 ^A,B,C,D,E,F^	2.16 ± 1.06 ^C^
	S	55.27 ± 2.15 ^D,E,F,G,H^	−5.65 ± 0.04 ^C,D,E^	25.93 ± 1.57 ^E,F,G^	7.71 ± 2.59 ^A,B,C^
Day 21	Uncoated	66.72 ± 0.10 ^B,C^	−6.37 ± 0.08 ^C,D,E^	32.29 ± 0.11 ^A,B,C,D^	
	T	64.97 ± 3.12 ^B,C,D^	−5.68 ± 0.40 ^C,D,E^	30.98 ± 1.29 ^A,B,C,D,E^	2.29 ± 1.32 ^C^
	S	54.57 ± 1.94 ^E,F,G,H^	−5.33 ± 0.70 ^C^	25.19 ± 2.12 ^E,F,G^	14.11 ± 2.78 ^A,B,C^
Day 42	Uncoated	70.35 ± 0.18 ^A,B^	−5.63 ± 0.05 ^C,D,E^	34.13 ± 0.28 ^A,B^	
	T	79.22 ± 6.75 ^A^	−2.10 ± 1.44 ^A^	35.23 ± 3.46 ^A^	9.61 ± 5.62 ^A,B,C^
	S	57.05 ± 5.97 ^C,D,E,F,G,H^	−3.53 ± 0.41 ^A,B^	28.95 ± 2.21 ^B,C,D,E,F,G^	14.43 ± 6.20 ^A,B^

* The data are mean ± SD of three replicates from two zucchinis. Different letters indicate significant differences with ANOVA and Tuckey post-hoc test at *p* < 0.05.

**Table 4:** Color evaluation of zucchini pulp during 42 days of storage.

Storage	Sample	L*	a*	b*	ΔE*
Day 1	Uncoated	82.09 ± 0.10 *^B^	−1.26 ± 0.04 ^A,B,C^	29.52 ± 0.06 ^E,F,G^	
	T	79.35 ± 2.62 ^B,C,D^	−3.52 ± 0.80 ^E^	31.21 ± 1.58 ^C,D,E,F^	3.94 ± 2.76 ^B,C^
	S	80.31 ± 0.94 ^B,C^	−2.91 ± 0.65 ^D,E^	30.46 ± 1.26 ^D,E,F,G^	2.60 ± 0.64 ^B,C^
Day 5	Uncoated	80.88 ± 0.25 ^B,C^	−1.32 ± 0.04 ^A,B,C^	30.91 ± 0.30 ^C,D,E,F,G^	
	T	80.60 ± 0.02 ^B,C^	−2.13 ± 0.06 ^C,D,E^	29.26 ± 0.91 ^E,F,G^	1.85 ± 0.76 ^B,C^
	S	77.80 ± 1.96 ^D^	−1.82 ± 1.48 ^B,C,D^	35.18 ± 0.10 ^B^	5.73 ± 1.42 ^B^
Day 12	Uncoated	81.05 ± 0.25 ^B,C^	−1.11 ± 0.05 ^A,B,C^	30.77 ± 0.09 ^C,D,E,F,G^	
	T	81.07 ± 0.87 ^B,C^	−1.18 ± 0.27 ^A,B,C^	29.03 ± 0.28 ^F,G^	1.74 ± 0.28 ^B,C^
	S	79.53 ± 1.27 ^B,C,D^	−0.80 ± 0.15 ^A,B,C^	33.02 ± 2.45 ^B,C,D^	2.74 ± 2.56 ^B,C^
Day 16	Uncoated	80.35 ± 0.25 ^B,C^	−0.84 ± 0.08 ^A,B,C^	31.46 ± 0.10 ^C,D,E,F^	
	T	80.09 ± 1.24 ^B,C,D^	−1.31 ± 0.54 ^A,B,C^	30.00 ± 2.03 ^D,E,F,G^	1.61 ± 1.27 ^B,C^
	S	78.80 ± 0.06 ^C,D^	−0.84 ± 0.50 ^A,B,C^	33.97 ± 0.51 ^B,C^	2.95 ± 0.39 ^B,C^
Day 21	Uncoated	80.73 ± 0.04 ^B,C^	−0.35 ± 0.02 ^A,B^	30.22 ± 0.09 ^D,E,F,G^	
	T	81.99 ± 0.41 ^B^	−0.84 ± 0.34 ^A,B,C^	27.74 ± 0.71 ^G^	2.83 ± 0.85 ^B,C^
	S	81.15 ± 0.63 ^B,C^	−1.02 ± 0.04 ^A,B,C^	30.00 ±1.75 ^D,E,F,G^	0.82 ± 0.77 ^C^
Day 42	Uncoated	86.64 ± 0.05 ^A^	−0.10 ± 0.03 ^A^	30.86 ± 0.07 ^C,D,E,F,G^	
	T	85.45 ± 0.21 ^A^	−0.49 ± 0.24 ^A,B^	32.37 ± 0.11 ^B,C,D,E^	1.96 ± 0.01 ^B,C^
	S	79.77 ± 1.12 ^B,C,D^	−1.50 ± 0.05 ^A,B,C,D^	39.48 ± 1.28 ^A^	11.11 ± 1.67 ^A^

* The data are mean ± SD of three replicates from two zucchinis. Different letters indicate significant differences with ANOVA and Tuckey post-hoc test at *p* < 0.05.

## Data Availability

Not applicable.

## Supplementary Materials

The following supporting information can be downloaded at: https://www.mdpi.com/article/10.3390/membranes12030326/s1, Figure S1: Rheological behavior during 3ITT test in small-amplitude oscillatory mode of obtained whey protein-thyme nanoemulsions. (a) T nanoemulsions (b) S nanoemulsions; Figure S2: Prooxidant effect on zucchinis; Table S1: Rheological parameters of T and S whey protein-thyme EO nanoemulsions compared to controls.
