# Supplementary material for: Efficacy of Two Stabilizers in Nanoemulsions with Whey Proteins and Thyme Essential Oil as Edible Coatings for Zucchini
Source: Membranes (Basel). 2022 Mar 15;12(3):326. doi: 10.3390/membranes12030326 (PMC8951633; doi:10.3390/membranes12030326)
Supplement: Supplementary file 1 [file membranes-12-00326-s001.zip › membranes-1613538-supplementary.pdf]

Supplementary Material

Efficacy of Two Stabilizers in Nanoemulsions with Whey Proteins and Thyme Essential Oil as Edible Coatings for Zucchini

Iulia Bleoanca [1], Andreea Lanciu [1], Livia Patrașcu [2], Alina Ceoromila [2] and Daniela Borda [1,*]

[1] Faculty of Food Science and Engineering, Dunarea de Jos University of Galati, 800201 Galati, Romania; Iulia.Bleoanca@ugal.ro (I.B.); andreealanciu@yahoo.com (A.L.)

[2] Cross-Border Faculty, Dunarea de Jos University of Galati, 111 Domneasca Str, 800201 Galati, Romania; Livia.Mantoc@ugal.ro (L.P.); Alina.Cantaragiu@ugal.ro (A.C.)

* Correspondence: Daniela.Borda@ugal.ro; Tel.: +40-336-130-177

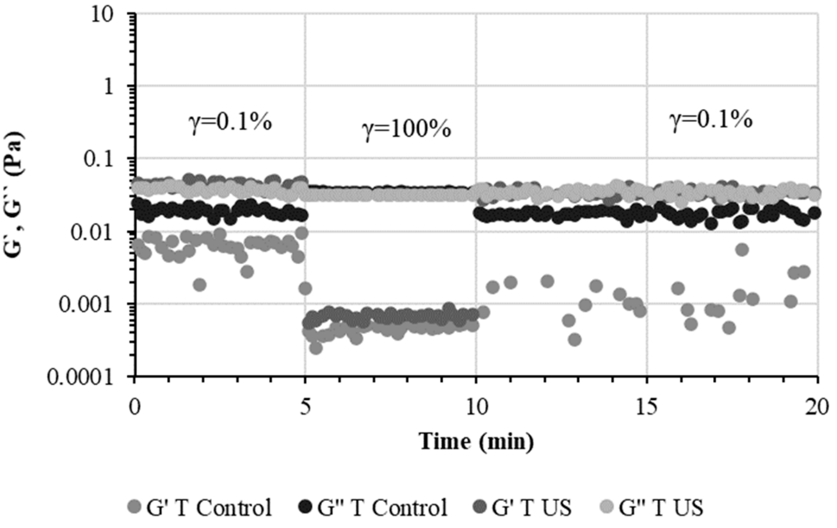

(**a**)

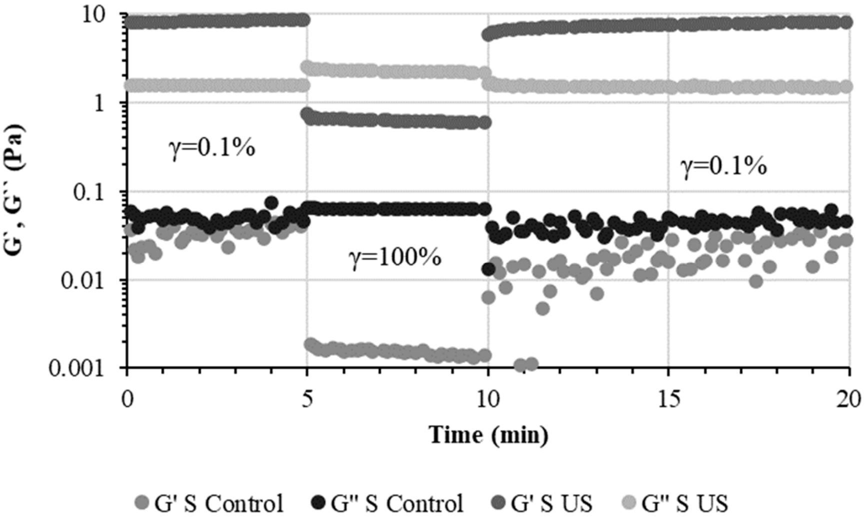

(**b**)

Figure S1. Rheological behavior during 3ITT test in small-amplitude oscillatory mode of obtained whey protein-thyme nanoemulsions. (**a**) T nanoemulsions; (**b**) S nanoemulsions.

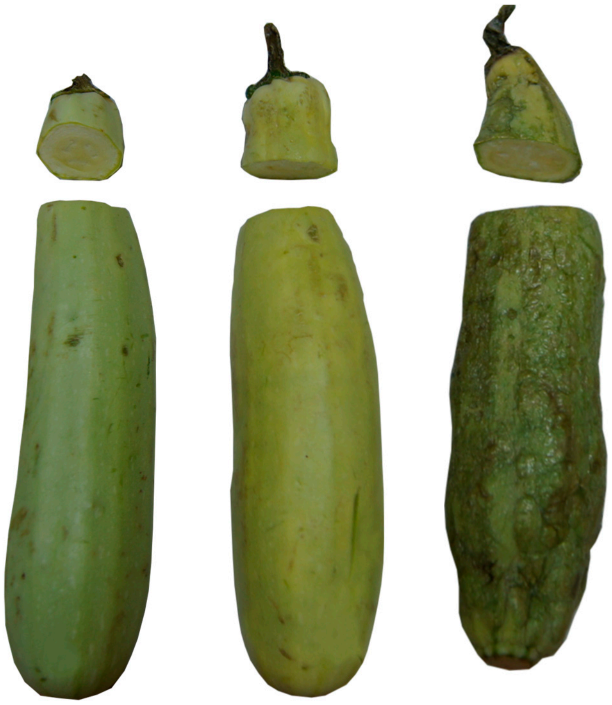

Figure S2. Prooxidant effect on zucchinis.

Table S1. Rheological parameters of T and S whey protein-thyme EO nanoemulsions compared to controls.

Sample	Apparent viscosity at 100 s^{-1} (mPa.s)	% strain at G′-G″ intersection
T US	4.66 ± 0.72 [a]	0.1171 ± 0.00 [a]
T Control	5.27 ± 0.01 [a]	0.0163 ± 0.00 [a]
S US	71.66 ± 1.85 [b]	25.2020 ± 0.24 [b]
S Control	9.77 ± 0.03 [a]	Viscous behavior with no intersection point

The data are mean ± SD of three replicates from two reading. Different letters indicate significant differences with ANOVA and Tuckey post- hoc test at $p < 0.05$.
